# The acceptability of using a lottery to allocate research funding: a survey of applicants

**DOI:** 10.1186/s41073-019-0089-z

**Published:** 2020-02-03

**Authors:** Mengyao Liu, Vernon Choy, Philip Clarke, Adrian Barnett, Tony Blakely, Lucy Pomeroy

**Affiliations:** 1 Health Research Council of New Zealand, Auckland, New Zealand; 2 Health Economics Research Centre, Nuffield Department of Population Health, University of Oxford, Oxford, UK; 3 Institute of Health and Biomedical Innovation & School of Public Health and Social Work, Queensland University of Technology, Brisbane, Queensland Australia; 4 Melbourne School of Population and Global Health, University of Melbourne, Melbourne, Victoria Australia

**Keywords:** Peer review, Research funding, Lottery

## Abstract

**Background:**

The Health Research Council of New Zealand is the first major government funding agency to use a lottery to allocate research funding for their Explorer Grant scheme. This is a somewhat controversial approach because, despite the documented problems of peer review, many researchers believe that funding should be allocated solely using peer review, and peer review is used almost ubiquitously by funding agencies around the world. Given the rarity of alternative funding schemes, there is interest in hearing from the first cohort of researchers to ever experience a lottery. Additionally, the Health Research Council of New Zealand wanted to hear from applicants about the acceptability of the randomisation process and anonymity of applicants.

**Methods:**

This paper presents the results of a survey of Health Research Council applicants from 2013 to 2019. The survey asked about the acceptability of using a lottery and if the lottery meant researchers took a different approach to their application.

**Results:**

The overall response rate was 39% (126 of 325 invites), with 30% (76 of 251) from applicants in the years 2013 to 2018, and 68% (50 of 74) for those in the year 2019 who were not aware of the funding result. There was agreement that randomisation is an acceptable method for allocating Explorer Grant funds with 63% (*n* = 79) in favour and 25% (*n* = 32) against. There was less support for allocating funds randomly for other grant types with only 40% (*n* = 50) in favour and 37% (*n* = 46) against. Support for a lottery was higher amongst those that had won funding. Multiple respondents stated that they supported a lottery when ineligible applications had been excluded and outstanding applications funded, so that the remaining applications were truly equal. Most applicants reported that the lottery did not change the time they spent preparing their application.

**Conclusions:**

The Health Research Council’s experience through the Explorer Grant scheme supports further uptake of a modified lottery.

## Background

Health and medical research aims to improve lives by using rigorous experiments to provide evidence to inform changes to policy and practice. This research often requires funding, and every year, billions of dollars of funding is awarded using competitive peer review, where researchers submit their project ideas and their peers aid the process to decide which ideas most deserve funding. This peer review system is used almost exclusively around the world [1], but there is very little scientific evidence that this is the best way to distribute scarce research dollars [2]. The paucity of research into research funding was noted by a recent literature search on grant peer review, which observed the need for, “open, transparent experimentation and evaluation of different ways to fund research” [3].

One alternative approach to allocating funding is to use lotteries or modified lotteries [4]. In a modified lottery, short applications are screened for eligibility and/or to remove weak applications, and then applications are funded at random until the budget is exhausted. This reduces the burden on peer reviewers and administrators, which is a concern as funders often struggle to find well-qualified reviewers within tight timelines [5]. This simplified approach also potentially reduces the burden on applicants, reviewers and funders if application forms can be simplified and if applicants reduce their preparation time because they recognise that funding is not guaranteed [6, 7]. This could return time and resources back to research given the large amount of time researchers spend on applications [8].

By reducing the role of people in decision making, lotteries also minimise the problems of sexism, racism and ageism influencing who receives funding [9]. Interestingly, lotteries may also increase fairness and support more meritorious ideas [10]. Lotteries also explicitly acknowledge the role of chance in winning funding, which occurs because the review process is somewhat random because of the selection and availability of peer reviewers [11].

Previous research found that amongst funded projects, the peer review score was a poor predictor of the subsequent number of research outputs [12]. Related recent research found a low agreement amongst reviewers scoring the same application [13]. Braben argued that the standard model of peer review will never accurately predict research success because it is inherently unpredictable [14]. This inherent unpredictability provides support for investigating lotteries as an alternative funding system.

Using lotteries to allocate research funding is a controversial idea to some, and can be viewed as “at odds with ingrained ideas about the meritocratic principles that govern the sciences” [15, 16]. It can be difficult to convince researchers and administrators that a lottery would be better than the “gold standard” of in-depth peer review [17].

The Health Research Council of New Zealand (HRC) was the first major funding agency to use a modified lottery to allocate research funding, starting in 2013 and continuing to the present day. Lotteries are used for a specific HRC funding scheme called the Explorer Grant project-funding scheme, which seeks to attract and fund transformative research ideas with the potential for major impact. Grants are available in any health research discipline. The scheme supports transformative research at an early stage and is designed for research that is not compatible with funding through other HRC schemes, which are not designed to provide support to explore potentially transformative research ideas at an early stage, as applications for greater investment through standard funding mechanisms require a clearly justified rationale and supporting data. Explorer Grants are fixed at NZD $150,000 (approximately USD $100,000) for up to 2 years. Individuals or groups are able to apply, as long as New Zealand is their principle domicile and place of employment. Explorer Grants account for 2% of the total annual funding expenditure. The number of applications and awards per year are shown in Table 1. The HRC considered random funding to be a fair and transparent approach to choose between equally qualified applicants and potentially particularly suited to Explorer Grants, where it may be difficult or even inappropriate to compare and score high-risk applications with unpredictable outcomes.

**Table 1:** Annual numbers of Explorer Grant applications and winners from 2013 to 2019

Year	Applications	Judged as ineligible by panel	Not funded by lottery	Funded by lottery
2013	116	99	14	3
2014	24	18	2	4
2015	45	38	3	4
2016	38	29	0	9
2017	34	21	2	11
2018	60	50	0	10
2019	77	53	9	15
Total	394	308	30	56

A modified lottery has also recently been adopted to allocate funding for a scheme administered by the Volkswagen Foundation in Germany [18] and the Swiss National Science Foundation [19].

In this paper we examine the results of a survey of Explorer Grant applicants conducted by the HRC. The survey asked about the acceptability of using a lottery for researchers, and if the random allocation meant researchers approached the application differently. The words “lottery” and “random” are both used to describe the funding allocation.

### The Explorer Grant assessment process

Applications are short (six pages with limited administrative information) and anonymised to the peer reviewers, thus directing the focus of assessment to the project idea while also potentially reducing bias and application costs.

The Explorer Grant assessment process has two steps. In the first step, eligible applications are assessed by one of four Explorer Grant Assessing Committee panels targeted to the nature of applications received. There are three broad panel areas of biomedical (two panels), clinical (one panel) and public health research (one panel). There are three members on each of the four panels with members selected based on a demonstrated ability to apply innovative thinking and approaches, demonstrated breadth of expertise, and with consideration to balance across the whole group of panel members (e.g., gender, location, age, institution).

Each application is rated by the three panel members for whether it meets the two criteria of being:

1. potentially transformative, which means has the potential to radically change the knowledge base or create a new paradigm or pathway, may be challenging to accept, is likely to be untested and is not a next step to current research or practice.
2. viable, which means the idea and methodology are potentially viable, the research environment is appropriate, and that sufficient progress can be made within the term of the grant.

Applications are anonymous, but panel members are asked to opt out of an assessment if they feel they recognise the applicants (New Zealand is a small country), and they are replaced by another non-conflicted panel member. Panel members give a “yes” or “no” assessment for each application and there are no additional external reviewers. Applications with two or more “yes” assessments enter the pool of fundable applications, whilst those that do not are declined. All applicants are informed of the outcome as being either “Declined”, “Fundable but not funded” or “Funded”.

In the second step, fundable applications are entered in Microsoft Excel and a random number is assigned using the Rand() function and applications are selected for funding up to the available budget in the order of smallest to largest random number.

The use of the lottery is clearly explained to all applicants in the documentation on how to apply. Applicants would also likely be aware of the lottery because of the high-profile international attention the scheme has received including journal articles [1], news stories and podcasts [20].

Applications are called for annually. Previous applicants are allowed to reapply regardless of any previous outcomes.

## Methods

### Survey

All 325 Explorer Grant applicants from the previous seven completed funding rounds (2013 to 2019) were emailed an invitation from the HRC to complete a short anonymous survey via *Survey Monkey* [21]*.* An automatic email reminder was sent to applicants with no response or partial response. The reminder was sent 9 days after first invite for the 2013 to 2018 survey and 11 days after first invite in the 2019 survey. The survey was designed to enable stakeholder feedback on the novel aspects of the Explorer Grant scheme, as part of a wider quality improvement project reviewing several aspects of investment processes to ensure practices are efficient and effective.

Applicants from the 2013 to 2018 funding rounds were emailed in September 2018, and applicants from the 2019 funding round were emailed in January 2019, with both surveys open for two weeks. Applicants were informed that the survey would be used to improve HRC processes and would be published in the public domain.

There was one open-ended and seven closed-ended questions, including questions about the randomisation process for allocating funding and the anonymity of applicants (see Additional file 1 for the survey questions). The survey questions were developed by the authors. Applicants were allowed to leave optional comments after all eight questions. Applicants had to complete all eight questions before submitting the survey; hence, there was no item-missing data. There were no incentives to participate.

Applicants from the 2013 to 2018 funding rounds were matched with their funding outcome. For applicants who had more than one application, they were represented once using their best outcome in the order of: Funded, Fundable and Declined. So we approached 325 applicants, whereas Table 1 shows 394 applications in total. The 2019 survey was conducted before the funding was awarded, and hence, the outcome data were not available for these respondents. There were ten applicants who were invited in both the 2018 and 2019 survey groups, and seven of them completed both of the 2018 and 2019 surveys. All responses were collected anonymously.

We summarise the results using frequency tables and bar plots. We include illustrative comments, whilst protecting anonymity, selected to include both positive and negative reactions.

We examined an association between the respondent’s funding outcome and their survey response for the two questions about the acceptability of randomisation (questions 2 and 3). We used a cross-tabulation and chi-squared test. From the response options of Yes, No and Unsure, we combined “No” and “Unsure” and compared them with “Yes”. Hence, we examined whether being funded was associated with a positive response to the use of randomisation. Because of small cell sizes in the table, we used a non-parametric permutation test to examine the independence of funding status and positive response to the use of randomisation using 1000 permutations [22].

We report our results using the Checklist for Reporting Results of Internet E-Surveys (CHERRIES) [23]. The tables and graphs were created using R version 3.6.0 [24].

## Results

The overall response rate was 126 respondents out of 325 invites (39%). The response rate was much higher for the most recent group of applicants in 2019 and for those who were funded (Table 2). All seven closed-ended questions received some additional comments, with a range from 10 to 41% and a median of 26%.

**Table 2:** Overall survey response rate and responses by time period and funding outcome

Time period* / Outcome	Response rate
2013 to 2018	30% (76 of 251)
Declined by the panel	24% (48 of 199)
Fundable but not funded by lottery	36% (5 of 14)
Funded by lottery	61% (23 of 38)
2019 (Outcome not available)	68% (50 of 74)

*All applicants of the 2013 to 2018 period were emailed in September 2018 (and were aware of the funding allocation), while the 2019 applicants were emailed in January 2019 (and were not aware of the allocation)

### Questions on random funding

**Fig. 1:**
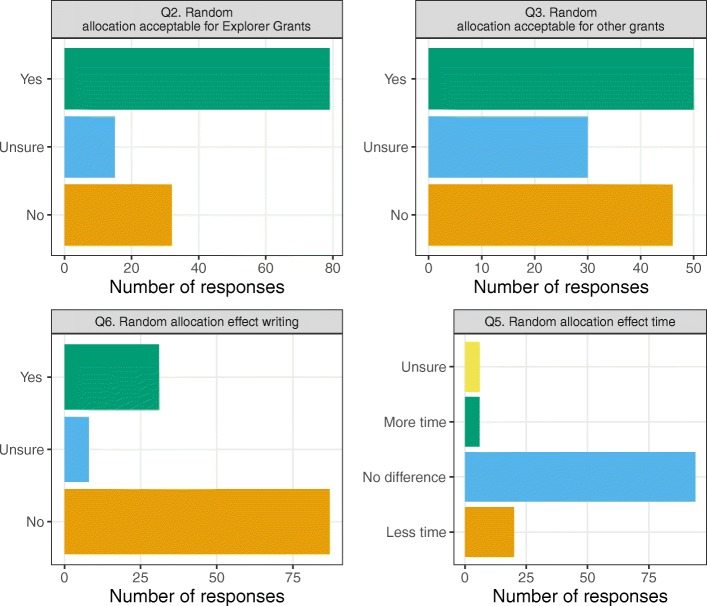
Bar charts of the responses to the four questions concerning the random allocation of funding (see Table 2 for complete question wording)

The results concerning the four questions on random funding are summarised in Fig. 1 and Table 3. There was agreement that randomisation is an acceptable method for allocating Explorer Grant funds with 63% (*n* = 79) positive. There was less support for allocating funds randomly for other grant types with only 40% (*n* = 50) positive and 37% (*n* = 46) negative, a close-to-perfect split in opinion.

**Table 3:** Responses to four questions concerning the random allocation of funding

Question number and text	Yes	Unsure		No
Q1. Was the format and length of the Explorer Grant application adequate for you to communicate the novelty and transformative nature of the proposal?	115 (91%)	6 (5%)		5 (4%)
Q2. Do you think the randomisation process is an acceptable method of allocating Explorer Grant funds?	79 (63%)	15 (12%)		32 (25%)
Q3. Do you think a randomisation process would be an acceptable method for the allocation of funding for other grant types?	50 (40%)	30 (24%)		46 (37%)
Q6. Did the knowledge that funding could be randomly allocated affect how you approached and/or wrote your Explorer Grant application?	31 (25%)	8 (6%)		87 (69%)
Q7. The identities of applicants are anonymous to the assessors. Do you think this is an acceptable approach for Explorer Grant assessment?	112 (89%)	12 (10%)		2 (2%)
	Less time	No difference	More time	Unsure
Q5. Did the knowledge that funding could be randomly allocated affect the amount of time you spent preparing your application?	20 (16%)	94 (75%)	6 (5%)	6 (5%)

Cells show the number and row percent (*N* = 126).

A number of respondents mentioned that random allocation was appropriate for Explorer Grants provided those applications that made it through the initial stage were “of equal merit”, “deemed worthy enough” or “reach the threshold requirements”. Hence, support for random allocation was conditional on the first step in the process creating a set of similar applications.

Some respondents were unsupportive of random allocation, including

“Why not just use the rank? Or judge on an [sic] potential impact score after screening?”

Others were more supportive, including

“I don't think a randomisation process is any less fair than an individual reviewer finding some minor reason for a great project not to be funded.”

Ten researchers mentioned similar concerns about the wider use of random allocation to other grant types in their comments, as they all put forward the idea of the “stand out applications” being funded and randomisation being used for the remaining fundable grants. Random allocation had greater support (beyond the Explorer Grants) if only applied to applications considered to be of comparable quality.

Most applicants said that the knowledge that funding could be randomly allocated made no difference to their approach to the application (*n* = 87, 69%) or to the amount of time they spent preparing the application (*n* = 94, 75%).

An applicant who thought they did change their approach said,

“I was focussed on making sure that I had clearly outlined how my proposed project met the transformative criteria and I knew project proposals weren't being ranked.”

An applicant who thought that the random allocation did not influence their time said,

“I try and give everything I do my best shot even when highly unlikely to succeed.”

The median time researchers estimated it took to prepare their application was 10 days (95% confidence interval for the median: 7 to 14 days). Retrospective questions about time spent are hard to complete accurately, and four applicants mentioned this difficulty in their comments.

### Questions on the application format and anonymous reviewers

Almost all applicants (*n* = 115, 91%) thought the format and length of the Explorer Grant application was adequate to communicate the novelty and transformative nature of the proposal (Table 3). There was strong support (*n* = 112, 89%) for the identity of applicants being anonymous to assessors (peer reviewers).

A number of respondents commented on the difficulty of making applications truly anonymous given the relatively small number of researchers in New Zealand (NZ). For example,

“For some grants there will be very few people in NZ who can write/are in the area. This means it may not be as anonymous as is intended.”

Others were supportive of anonymity:

“I really like the anonymous part of it as this allows the ideas to shine and not the track record of the researchers.”

### Association between funding outcome and survey response

Respondents who had won funding were far more positive about the use of random funding allocation (Table 4). Seventy-eight percent of respondents who had won Explorer Grant funding thought randomisation was acceptable, compared with 44% for those whose applications were declined by the panel. Similarly, far more applicants who had won funding supported an expansion of random funding into other grant types.

**Table 4:** Associations between funding outcome and a positive response to the questions on the acceptability of randomisation

Question number and text	Funded by lottery *n* / *N*	Not funded by lottery *n* / *N*	Declined by panel *n* / *N*	*p* value
Q2. Do you think the randomisation process is an acceptable method of allocating Explorer Grant funds?	18 / 23 (78%)	3 / 5 (60%)	21 / 48 (44%)	0.044
Q3. Do you think a randomisation process would be an acceptable method for the allocation of funding for other grant types?	13 / 23 (57%)	0 / 5 (0%)	12 / 48 (25%)	0.010

Cells are the number of positive responses / total number of responses, and the percent of positive responses. Surveys from applicants in 2013 to 2018 (*N* = 76). The last column is the *p* value from a permutation test of the independence between funding outcome and positive response to the questions.

## Discussion

The New Zealand HRC Explorer Grant scheme is the world’s first scheme to allocate government funding for scientific research at random [1]. It has been using random funding since 2013 and has grown in size with the most recent round allocating five times as many grants as the original round.

Twenty-five percent of survey respondents thought that randomisation is inappropriate for Explorer Grants (Table 2); hence, there is support for randomisation amongst this first-ever cohort to experience it. Whilst there was support for randomisation being an acceptable method for Explorer Grants, there was less support for randomising other grant types. This difference may be linked to the nature of Explorer Grants which have a smaller budget than other schemes and specifically target more risky innovative projects. The outcomes from potentially transformative research are almost impossible to predict, and Ioannidis argued that a lottery is a logical approach to funding that could save time and would better spread funding across more researchers [25]. For larger grants, there may be more support for funding “stand out” applications and randomly allocating funding to applications in the grey zone between “not fundable” and “outstanding”.

Support for randomisation was higher amongst researchers who won funding, which indicates the difficulty of decoupling researchers’ thoughts about a funding system from their personal experience, especially given the pressure to win funding and the potential impacts on researchers’ careers [26]. Support for a lottery, or any funding system, is likely to be higher when success rates are high.

A surprising result was that most applicants did not reduce the amount of time they spent on the application. A likely explanation, highlighted in a number of comments in the survey, is that the applications had to pass an initial peer review stage to make it into the lottery; hence, researchers still need to convince their peers of the project’s merits. One of the purported advantages of lotteries has been the time they would save for researchers, because applications could be greatly shortened [6, 7]. This advantage may have been over-stated, and may only apply where there is little or no peer review. A related finding comes from an Australian study of grant application forms, where a reduction in the length of forms did not reduce application times [27]. The time costs for applicants may predominantly be in the process of sculpting and expressing their key ideas. As one applicant commented, “I am pretty excited about this project and so, in the end, it [the random allocation] had no impact on the effort I put into preparing my bid.” It is also possible that while researchers do not reduce preparation time in the initial application, they can recycle unsuccessful lottery applications without having to spend time modifying them.

We are not aware of any similar surveys of researchers. A previous survey of Australian researchers did ask a hypothetical question about a funding lottery. Researchers were asked if their proposal was ranked by a peer review panel as “Possibly Fund”, if they would accept a lottery draw amongst the “Possibly Fund” proposals, and 43% agreed [28]. This is close to the 40% agreement from this survey for using a lottery for other grant schemes (Table 2).

Support for the identity of applicants being anonymous to assessors was high, with the majority of comments recognising this reduces the potential for bias. Anonymising applicant identities to assessors is one of a number of steps to minimise the influence of bias, including ensuring committee membership is diverse, giving visibility to the issue of bias and other actions to support quality decision making [29].

### Limitations

There are some limitations to this study. The response rate was 39%, and we had a better response from those who were funded. Hence, our sample likely over-represents those who are happier with the system.

An interesting comparison would be between researchers who entered the lottery and were funded or not (Funded vs Fundable). However, the numbers in the Fundable group were relatively small and any statistical comparison would likely have large uncertainty.

We cannot track how perceptions may have changed over time, and there is potential recall bias given for the applicants from 2013 to 2018 as they were surveyed in 2018. Also, the funding success rate during this period was low (14%), and this likely influenced responses as we found an association between funding outcomes and attitudes to randomisation.

We did not ask the peer reviewers whether they spent less time reviewing the applications, and this is another potential source of cost savings for lotteries compared with standard peer review systems. Reviewers may need less time to put applications into categories compared with the standard task of ranking each application without ties. A study in Australia compared a linear ranking system with three categories (should fund, could fund and should not fund) and found that the two panels “with almost complete consensus” believed that the categorising vastly improved the peer review process [30].

We did not use a formal qualitative analysis of the applicants’ comments, but instead selected comments that we felt illustrated common themes.

The survey was sent to applicants by the New Zealand Health Research Council, and this may have influenced applicants’ responses despite the survey being clearly labelled as anonymous. An ideal future study would be independently run and would compare across funding agencies and schemes.

## Conclusions

Health research funders have significant responsibility for investment decisions that have the potential to improve health outcomes. Innovation in health research funding processes, as an integrated part of the wider health research sector, can add to the value, impact and sustainability of the sector. The HRC’s experience through the Explorer Grant scheme supports further uptake of a modified lottery.

## Supplementary information

**Additional file 1.** Survey questions.

## Data Availability

The datasets generated and/or analysed during the current study are not publicly available because consent was not obtained for the data’s wider use.

## Abbreviations

HRC: Health Research Council
NZ: New Zealand
