# Supplementary material for: The acceptability of using a lottery to allocate research funding: a survey of applicants
Source: Res Integr Peer Rev. 2020 Feb 3;5:3. doi: 10.1186/s41073-019-0089-z (PMC6996170; doi:10.1186/s41073-019-0089-z)
Supplement: Supplementary file 1 — Additional file 1. Survey questions. [file 41073_2019_89_MOESM1_ESM.docx]

**Additional file 1: Survey questions**

Q1. Was the format and length of the Explorer Grant application adequate for you to communicate the novelty and transformative nature of the proposal?

Q2. Do you think the randomisation process is an acceptable method of allocating Explorer Grant funds?

Q3. Do you think a randomisation process would be an acceptable method for the allocation of funding for other grant types?

Q4. How long did it take you to prepare your application? Please give us an estimate of your time in total working days.

Q5. Did the knowledge that funding could be randomly allocated affect the amount of time you spent preparing your application?

Q6. Did the knowledge that funding could be randomly allocated affect how you approached and/or wrote your Explorer Grant application?

Q7. The identities of applicants are anonymous to the assessors. Do you think this is an acceptable approach for Explorer Grant assessment?

Q8. Do you have any comments on how the concept of transformative research applies to health, and/or any suggestions about how the HRC could improve our guidance on transformative research?

Questions 1 to 7 were followed by a space for optional comments.

**Additional table 1: Number of comments per question**

Number and percent of comments received per question.

| Question | n | Percent |
| --- | --- | --- |
| Q1. Was the format and length of the Explorer Grant application adequate for you to communicate the novelty and transformative nature of the proposal? | 12 | 10 |
| Q2. Do you think the randomisation process is an acceptable method of allocating Explorer Grant funds | 38 | 30 |
| Q3. Do you think a randomisation process would be an acceptable method for the allocation of funding for other grant types? | 52 | 41 |
| Q4. How long did it take you to prepare your application? Please give us an estimate of your time in total working days | 33 | 26 |
| Q5. Did the knowledge that funding could be randomly allocated affect the amount of time you spent preparing your application? | 22 | 17 |
| Q6. Did the knowledge that funding could be randomly allocated affect how you approached and/or wrote your Explorer Grant application? | 26 | 21 |
| Q7. The identities of applicants are anonymous to the assessors. Do you think this is an acceptable approach for Explorer Grant assessment? | 45 | 36 |

**Additional table 2: Responses split by survey time**

| q | survey.nice | No | Unsure | Yes |
| --- | --- | --- | --- | --- |
| 1 | 2013-2018 | 5 (7%) | 5 (7%) | 66 (87%) |
| 1 | 2019 | 0 (0%) | 1 (2%) | 49 (98%) |
| 2 | 2013-2018 | 26 (34%) | 8 (11%) | 42 (55%) |
| 2 | 2019 | 6 (12%) | 7 (14%) | 37 (74%) |
| 3 | 2013-2018 | 33 (43%) | 18 (24%) | 25 (33%) |
| 3 | 2019 | 13 (26%) | 12 (24%) | 25 (50%) |
| 6 | 2013-2018 | 52 (68%) | 6 (8%) | 18 (24%) |
| 6 | 2019 | 35 (70%) | 2 (4%) | 13 (26%) |
| 7 | 2013-2018 | 2 (3%) | 6 (8%) | 68 (89%) |
| 7 | 2019 | 0 (0%) | 6 (12%) | 44 (88%) |
